# Perioperative outcomes of penile prosthesis implantation in Germany: results from the GRAND study

**DOI:** 10.1038/s41443-023-00796-0

**Published:** 2023-11-18

**Authors:** Nikolaos Pyrgidis, Gerald B. Schulz, Michael Chaloupka, Yannic Volz, Paulo L. Pfitzinger, Severin Rodler, Elena Berg, Philipp Weinhold, Friedrich Jokisch, Christian G. Stief, Armin J. Becker, Julian Marcon

**Affiliations:** Department of Urology, University Hospital, LMU Munich, Munich, Germany

**Keywords:** Erectile dysfunction, Clinical trial design

## Abstract

We aimed to assess the recommended annual hospital volume for inflatable penile prosthesis implantation (PPI) and to provide evidence on perioperative outcomes of semi-rigid and inflatable PPI in Germany. We used the GeRmAn Nationwide inpatient Data (GRAND) from 2005 to 2021 and report the largest study to date with 7,222 patients. 6,818 (94.4%) patients underwent inflatable and 404 (5.6%) semi-rigid PPI. Inflatable PPI was significantly associated with shorter length of hospital stay (difference of 2.2 days, 95%CI: 1.6–2.7, *p* < 0.001), lower odds of perioperative urinary tract infections (5.5% versus 9.2%; OR: 0.58, 95%CI: 0.41–0.84, *p* = 0.003) and surgical wound infections (1% versus 2.5%; OR: 0.42, 95%CI: 0.22–0.88, *p* = 0.012) compared to semi-rigid PPI. Overall, 4255 (62.4%) inflatable PPIs were undertaken in low- ( < 20 PPI/year) and 2563 (37.6%) in high-volume ( ≥ 20 PPI/year) centers. High-volume centers were significantly associated with shorter length of hospital stay (difference of 1.4 days, 95%CI: 1.2–1.7, *p* < 0.001) compared to low-volume centers. Our findings suggest that inflatable PPI leads to a shorter length of hospital stay and lower rates of perioperative urinary tract and surgical wound infections compared to semi-rigid PPI. Patients undergoing surgery in high-volume centers for inflatable PPI are discharged earlier from the hospital.

## Introduction

Penile prosthesis implantation (PPI) is considered the gold standard treatment in patients with refractory erectile dysfunction (ED). Penile implants are typically categorized into inflatable and semi-rigid devices [1]. Semi-rigid or malleable devices result in a firm penis that can be manually bent into an erect or flaccid state. They are associated with reduced material costs, can be easily used by patients, and are recommended for those with limited manual dexterity and strength [2–4]. On the other hand, semi-rigid devices result in unnatural and persistent erections. Therefore, it seems that most patients prefer inflatable devices due to their “natural” erections and their concealability and comfort [5, 6]. Approximately 15,000 PPIs are performed worldwide every year. Of them, more than 85% of PPIs are carried out in the USA, with Germany contributing the second most implants in the world with about 2.5% of all cases [7]. In the USA, the proportion of inflatable PPI cases every year is about 10:1 compared to semi-rigid PPI cases [8].

PPI should be performed by prosthetic surgeons with an adequate caseload volume of at least one PPI/month [9, 10]. Even though PPI has evolved as a safe and widely used treatment for ED, it has been postulated that inflatable PPI might be associated with higher morbidity and prolonged hospital stay compared to semi-rigid PPI [11, 12]. Still, nationwide, high-volume studies directly comparing perioperative outcomes after inflatable versus semi-rigid PPI are scarce [13, 14]. Accumulating evidence suggests that increased annual hospital volume for inflatable PPI might be associated with improved perioperative outcomes [15]. High-volume hospitals provide, in most cases, the necessary infrastructure and trained medical and paramedical staff. Additionally, experienced operative teams can shorten the operative time, reduce postoperative complications, and improve the management of most intraoperative complications [16]. Thus, inflatable PPI is moving more and more towards centralization [17].

Studies from the USA have attempted to identify an annual hospital and surgeon volume threshold for PPI cases that might improve perioperative outcomes [9, 15, 17]. Nevertheless, the optimal annual caseload volume for inflatable PPI outside the USA remains unknown [18]. Moreover, studies exploring the current trends and outcomes after PPI in Germany are lacking [19]. Within this framework, we aimed to assess the recommended annual hospital volume for inflatable PPI and to provide evidence on the current trends and perioperative outcomes of semi-rigid and inflatable PPI in Germany through the largest study in the field.

## Methods

### GeRmAn Nationwide inpatient Data (GRAND)

All inpatient data in Germany from 2005 to 2021 are stored anonymized at the Research Data Center of the Federal Bureau of Statistics. They were retrieved for further analyses after agreement (LMU - 4710-2022) through the GRAND study. The GRAND study contains all inpatient cases in Germany except for psychiatric, and military cases, as well as those involving forensic medicine. Since 2005, all hospitals are required to transfer patient data on coexisting conditions, inpatient diagnoses, and procedures, as well as on perioperative outcomes to the German Institute for the Hospital Remuneration System to receive their remuneration. These patient data are coded based on the International Statistical Classification of Diseases and Related Health Problems, 10th revision, German modification (ICD-10-GM), and the German Procedure Classification (OPS) [20]. To ensure consistent documentation in Germany, coding guidelines are published by the German Institute for Medical Documentation and Information.

It should be highlighted that some important in-hospital information such as patients’ laboratory findings, operative time, as well as long-term complications and functional outcomes are not available in the GRAND database. Similarly, information on morbidity after hospital discharge, readmission and reoperation rates, satisfaction rates, and further follow-up data are also not included in this database.

### Outcomes

For the present analysis from the GRAND study, we included all patients undergoing PPI for the first time (OPS code for semi-rigid: 5-649.50 and inflatable PPI: 5-649.51). Studies from the USA have defined high-volume surgeons as those who perform at least one inflatable PPI per month [9]. Based on the previous notion, they consider high-volume centers, as those that perform at least 20 inflatable PPI cases/year [9, 15]. Therefore, all hospitals performing inflatable PPI in Germany were identified through their postal code and were further categorized based on their annual caseload volume to low- ( < 20 inflatable PPI cases/year), and high-volume centers ( ≥ 20 inflatable PPI cases/year) [15].

The primary outcome of the study was to compare low- versus high-volume centers in terms of important perioperative complications including sepsis, mortality, intensive care unit (ICU) admission, urinary tract infection, and surgical wound infection, as well as length of hospital stay. Secondary outcomes included the comparison of perioperative complications and length of hospital stay between semi-rigid and inflatable PPI, as well as the evolution of PPI in Germany through the last years.

### Statistical analysis

All categorical variables were reported as frequencies with proportions and compared with the chi-squared test. All continuous variables are normally distributed, reported as mean ± standard deviation (SD) and compared with the t-test. We performed multiple multivariable logistic and linear regression analyses to assess the role of annual hospital caseload volume of inflatable PPI on perioperative outcomes (complications and length of hospital stay), as well as to compare perioperative outcomes of semi-rigid versus inflatable PPI. All regression models were adjusted for age, obesity, diabetes, and prior pelvic radiation therapy. Odds ratios (ORs) with 95% confidence intervals (CIs) were provided for all logistic models and two-sided p-values lower than 0.05 were considered statistically significant.

Our research team did not have direct access to patient-level data but only to summary results provided by the Research Data Center of the Federal Bureau of Statistics. Therefore, all analyses were performed, on our behalf, from the Research Data Center based on R codes developed by our research team (source: Research Data Center of the Federal Bureau of Statistics, Diagnosis Related Groups -DRG- Statistics 2005-2021, own calculations). Patients’ baseline characteristics and in-hospital complications with fewer than three measures were not included in the summary results provided by the Research Data Center to ensure anonymity. Based on the previous notion, approval by an ethics committee or patient informed consent was not mandatory following the German legislation.

## Results

### Baseline characteristics of inflatable versus semi-rigid PPI

A total of 7,222 patients with a mean age of 52 ± 15 years underwent either inflatable (*n* = 6818, 94.4%) or semi-rigid (*n* = 404, 5.6%) PPI in Germany from 2005 to 2021. Of them, 1547 (21%) patients had diabetes, 563 (7.8%) suffered from obesity (body mass index > 30 kg/m^2^) and 2283 (32%) from hypertension. Patients with priapism as a cause of ED more frequently received semi-rigid PPI (*p* = 0.002). On the contrary, patients receiving inflatable PPI were older (*p* < 0.001) and more often had hypertension (*p* = 0.002). Both groups displayed similar rates of perioperative acute kidney disease and blood transfusion. The baseline characteristics of the included patients are presented in Table 1.

**Table 1:** Baseline characteristics of patients undergoing inflatable and semi-rigid PPI.

Characteristic	Overall, *n* = 7222	Inflatable PPI, *n* = 6818	Semi-rigid PPI, *n* = 404	*p*-value
**Age (years)**	52 ± 15	52 ± 15	49 ± 16	**<0.001**
**Diabetes**	1547 (21%)	1469 (22%)	78 (19%)	**0.32**
**Chronic kidney disease**	261 (3.6%)	245 (3.6%)	16 (4%)	**0.81**
**Hypertension**	2283 (32%)	2184 (32%)	99 (25%)	**0.002**
**Obesity**	563 (7.8%)	526 (7.7%)	37 (9.2%)	**0.34**
**Peyronie’s disease**	424 (5.9%)	409 (6%)	15 (3.7%)	**0.073**
**Priapism**	47 (0.7%)	39 (0.6%)	8 (2%)	**0.002**
**Prior pelvic radiation**	192 (2.7%)	187 (2.7%)	5 (1.2%)	**0.10**
**Acute kidney disease**	12 (0.2%)	12 (0.2%)	0 (0%)	**0.83**
**Transfusion**	22 (0.3%)	22 (0.3%)	0 (0%)	**0.50**

Variables are presented as mean ± standard deviation or frequencies with proportions. The *t*-test was performed for comparisons between continuous variables and the chi-squared test for categorical variables. The bold cells indicate statistically significant *p*-values. Categorical variables were compared with the chi-squared test and continuous variables with the *t*-test.

*PPI* Penile prosthesis implantation.

**Fig. 1:**
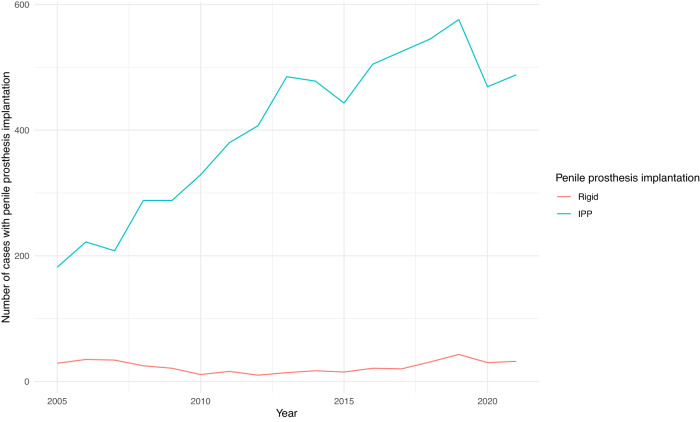
The annual number of cases of penile prosthesis implantation. IPP Inflatable penile prosthesis.

The number of patients undergoing inflatable PPI in Germany substantially increased in the last years reaching a maximum of 576 yearly cases in 2019 (from 182 cases in 2005). On the contrary, the number of patients undergoing semi-rigid PPI remained relatively stable from 29 cases in 2005 to 43 cases in 2019. Interestingly, the COVID-19 pandemic led to an important decrease in both inflatable and semi-rigid PPI cases. In particular, 469 patients received inflatable and 30 semi-rigid PPI in 2020. Accordingly, 488 patients received inflatable and 32 semi-rigid PPI in 2021. The annual trends of PPI are depicted in Fig. 1.

### Perioperative outcomes of inflatable versus semi-rigid PPI

The mean length of hospital stay was 7.4 ± 5 days for patients undergoing inflatable PPI and 9.7 ± 9 days for patients undergoing semi-rigid PPI. In the multivariate analysis after adjusting for age, obesity, diabetes, and prior pelvic radiation therapy, inflatable PPI was significantly associated with shorter length of hospital stay (difference of 2.2 days, 95% CI: 1.6–2.7, *p* < 0.001) compared to semi-rigid PPI. Similarly, inflatable PPI was associated with significantly lower odds of perioperative urinary tract infections (5.5% versus 9.2%; OR: 0.58, 95% CI: 0.41–0.84, *p* = 0.003) and surgical wound infections (1% versus 2.5%; OR: 0.42, 95% CI: 0.22–0.88, *p* = 0.012). On the contrary, the odds of ICU admission (0.7% versus 1%; OR: 0.67, 95% CI: 0.27–2.2, *p* = 0.4) did not differ between the two groups. Importantly, in-hospital mortality and sepsis could not be assessed, since in each group fewer than three patients developed these complications and the measures were excluded by the Research Data Center to ensure anonymity. All analyses are available in Table 2.

**Table 2:** Multivariable logistic and linear regression analysis comparing length of hospital stay, ICU admission rates, urinary tract infection rates and wound infection rates after semi-rigid versus inflatable PPI.

	Hospital stay			ICU admission			Urinary tract infection			Surgical wound infection		
PPI	Days	Beta	*p*-value	Events	OR	*p*-value	Events	OR	*p*-value	Events	OR	*p*-value
Semi-rigid	9.7 ± 9	—		4 (1%)	—		37 (9.2%)	—		10 (2.5%)	—	
Inflatable	7.4 ± 5	−2.2 (−2.7, −1.6)	**<** **0.001**	46 (0.7%)	0.67 (0.27, 2.2)	0.4	372 (5.5%)	0.58 (0.41, 0.84)	**0.003**	70 (1%)	0.42 (0.22, 0.88)	**0.012**

All models are adjusted for age, obesity, diabetes, and prior radiation therapy. The bold cells indicate statistically significant *p*-values. All logistic regression models were adjusted for age, obesity, diabetes, and prior pelvic radiation therapy.

*ICU* Intensive care unit, *OR* Odds ratio, *PPI* Penile prosthesis implantation.

### Baseline characteristics of inflatable PPI based on hospital caseload

**Fig. 2:**
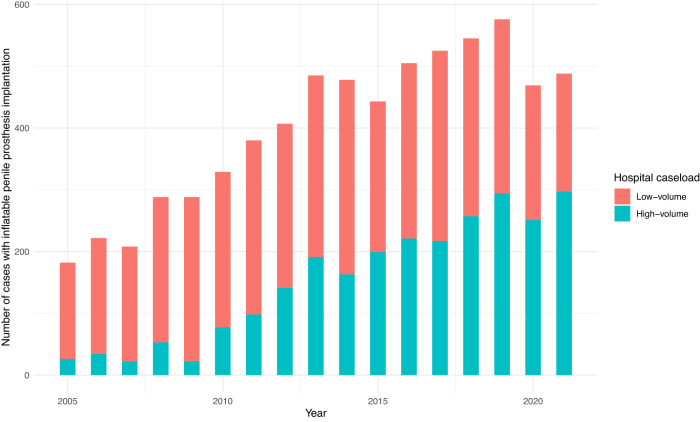
The annual trends for penile prosthesis implantation based on hospital caseload.

A total of 4,255 (62.4%) inflatable PPIs were undertaken in low- and 2563 (37.6%) in high-volume centers. The number of patients who underwent inflatable PPI in a high-volume center in Germany presented a substantial increase in the last years. Patients operated in low-volume centers were older and presented a significantly higher proportion of diabetes, chronic kidney disease, and hypertension compared to high-volume centers (*p* < 0.001 for all characteristics). Moreover, the proportion of patients requiring inflatable PPI due to priapism was also higher in low-volume centers (*p* = 0.002). The characteristics of all patients undergoing inflatable PPI and the annual trends of inflatable PPI based on the annual hospital caseload are provided in Table 3 and Fig. 2.

**Table 3:** Baseline characteristics of patients undergoing inflatable penile prosthesis implantation based on the suggested annual hospital caseload.

Characteristic	Low-volume centers (< 20 cases/year) *n* = 4255	High-volume centers (≥ 20 cases/year) *n* = 2563	*p*-value
Age (years)	56 ± 12	46 ± 16	**<0.001**
Diabetes	1102 (26%)	367 (14%)	**<0.001**
Chronic kidney disease	193 (4.5%)	52 (2%)	**<0.001**
Hypertension	1570 (37%)	614 (24%)	**<0.001**
Obesity	339 (8%)	187 (7.3%)	0.34
Peyronie’s disease	271 (6.4%)	138 (5.4%)	0.11
Priapism	34 (0.8%)	5 (0.2%)	**0.002**
Prior pelvic radiation	118 (2.8%)	69 (2.7%)	0.90
Acute kidney disease	9 (0.2%)	3 (0.1%)	0.55
Transfusion	16 (0.4%)	6 (0.2%)	0.44

Variables are presented as mean ± standard deviation or frequencies with proportions. The *t*-test was performed for comparisons between continuous variables and the chi-squared test for categorical variables. The bold cells indicate statistically significant *p*-values. Categorical variables were compared with the chi-squared test and continuous variables with the *t*-test.

### Perioperative outcomes of inflatable PPI based on hospital caseload

The mean length of hospital stay was 6.9 ± 3.3 days for patients undergoing inflatable PPI in high-volume centers and 7.7 ± 5.8 days for those undergoing surgery in low-volume centers. Upon multivariate analysis after adjusting for age, obesity, diabetes, and prior radiation therapy, high-volume centers were significantly associated with shorter length of hospital stay (difference of 1.4 days, 95% CI: 1.2–1.7, *p* < 0.001) compared to low-volume centers. On the contrary, the odds of perioperative ICU admission (0.5% versus 0.8%; OR: 0.78, 95% CI: 0.39–1.5, *p* = 0.5), surgical wound infection (0.9% versus 1.1%; OR: 0.74, 95% CI: 0.43–1.3, *p* = 0.3) and urinary tract infection (5.9% versus 5.2%; OR: 1.1, 95% CI: 0.84–01.3, *p* = 0.6) did not differ between high- and low-volume centers. In-hospital mortality and sepsis could not be assessed due to fewer than three complications in the whole cohort. All analyses are illustrated in Table 4.

**Table 4:** Multivariable logistic and linear regression analysis for the effect of annual hospital inflatable PPI caseload on length of hospital stay, ICU admission rates, urinary tract infection rates and wound infection rates.

	Hospital stay			ICU admission			Urinary tract infection			Surgical wound infection		
Volume	Days	Beta	*p*-value	Events	OR	*p*-value	Events	OR	*p*-value	Events	OR	*p*-value
<20	7.7 ± 5.8	—		33 (0.8%)	—		221 (5.2%)	—		47 (1.1%)	—	
≥20	6.9 ± 3.3	−1.4 (−1.7, −1.2)	**<0.001**	13 (0.5%)	0.78 (0.39, 1.5)	0.5	151 (5.9%)	1.1 (0.84, 1.3)	0.6	23 (0.9%)	0.74 (0.43, 1.3)	0.3

*ICU* Intensive care unit, *OR* odds ratio, *PPI* Penile prosthesis implantation.

All models are adjusted for age, obesity, diabetes, and prior radiation therapy. The bold cells indicate statistically significant *p*-values. All linear regression models were adjusted for age, obesity, diabetes, and prior pelvic radiation therapy.

## Discussion

The findings of the present study demonstrate that PPI is a safe surgical solution with low rates of perioperative complications in patients with refractory ED. Based on our analyses, inflatable PPI was associated with shorter length of hospital stay, as well as lower rates of urinary tract and surgical wound infections compared to semi-rigid PPI. Accordingly, the length of hospital stay was also shorter in high-volume centers performing inflatable PPI. The German nationwide data from 2005 to 2021 indicate that inflatable PPI is preferred in most patients with refractory ED. Inflatable PPI has undergone a 3-fold increase in the last years, whereas semi-rigid PPI has remained stable. Interestingly, the COVID-19 pandemic led to a decrease of approximately 20% in annual PPI cases compared to the pre-pandemic years.

Awareness regarding major perioperative complication rates after PPI is critical both for the prosthetic surgeon to improve the safety of the procedure and for the patients to be adequately counseled in the preoperative setting. Single-center studies from the USA have reported postoperative scrotal hematoma rates of about 3% [21] and surgical wound infection rates of about 1% [22]. Nevertheless, it should be highlighted that studies focusing on perioperative outcomes are scarce and that German nationwide multicenter data on the matter have previously not been described in the literature. Accordingly, no high-volume, real-world data on the in-hospital rates of urinary tract infection, sepsis, ICU admission, and mortality exist. Moreover, in a field of research where head-to-head comparative studies assessing inflatable versus semi-rigid PPI are lacking, the present analysis not only suggests that most patients and surgeons prefer inflatable devices but also that inflatable devices are associated with better perioperative outcomes.

It has been postulated that the acquaintance of each prosthetic surgeon with inflatable PPI plays a crucial role in the long-term outcomes. PPI in centers of excellence has been associated with shorter operative time and better long-term surgical outcomes. Data suggests that patients undergoing PPI in high-volume centers present lower revision rates and fewer surgical complications [23]. Nevertheless, our analyses suggest that surgery in high-volume centers does not lead to better perioperative outcomes. Increased annual hospital caseload seems only to shorten the length of hospital stay by about 1.5 days. It should be stressed that patients are typically discharged on the first postoperative day in the USA and on the third postoperative day in Germany after a complication-free inflatable PPI [24, 25]. Nevertheless, it should be noted that, in many cases, patients in Germany display a prolonged hospital stay. The latter is mainly explained by the fact that the German healthcare insurances cover the in-hospital costs in accordance with the DRG system. Thus, clinicians may prolong the patients’ hospital stay to ensure optimal perioperative care [26].

Shortly after the outbreak of the COVID-19 pandemic crisis, major urological guidelines classified surgeries in sexual medicine, such as PPI, as elective, low-priority surgeries and recommended postponing them [27]. This postponement was mandatory to prioritize the management of oncological and urologic emergency cases and to provide the necessary capacity and support for patients with COVID-19 [28, 29]. Nevertheless, based on our analyses, it seems that the German healthcare system could adequately compensate this postponement without backlogging after the strict COVID-19 pandemic waves. Most PPI surgeries were soon rescheduled and only a 20% decrease in the annual PPI cases was observed in 2020 and 2021, indicating that the German healthcare system recovered relatively promptly after the COVID-19 lockdown period.

Although we report, to our knowledge, the largest analysis on PPI, the present findings should be interpreted in the context of some important limitations. All findings were based on retrospective, administrative data, and, thus, are prone to coding errors, although these data present a relatively high degree of accuracy since they are controlled by independent physician task forces. Importantly, information on postoperative care, catheter, or drainage placement, preferred surgical approach, type, or duration of postoperative antibiotic administration are not included in the GRAND. Accordingly, the short- and long-term rates of device infection and mechanical failure are also not part of the present database. Similarly, information on annual surgeon’s caseload volume and outcomes could not be retrieved. Of note, all outcomes derive from Germany and, therefore, they may not be extrapolated to other healthcare systems. Still, in an attempt to overcome these limitations, our holistic approach through multiple analyses based on high-volume data may serve predominantly as a valuable guide for proper patient counseling and for the design and implementation of high-quality studies in the field.

## Conclusion

The present real-world data demonstrate that placement of an inflatable PPI leads to shorter length of hospital stay and lower rates of perioperative urinary tract and surgical wound infections compared to semi-rigid PPI. Patients undergoing surgery in high-volume centers for inflatable PPI are discharged earlier from the hospital, but the perioperative outcomes between high- and low-volume centers seem to be comparable. Of note, inflatable PPI is preferred in most German patients and has undergone a 3-fold increase in the last 17 years. Accordingly, despite the postponement of PPI during the COVID-19 pandemic crisis, the annual PPI cases displayed only a 20% decrease in 2020 and 2021 compared to the years before the COVID-19 pandemic. Overall, data from the GRAND study highlights that both the semi-rigid and the inflatable PPI should be considered a safe and well-established solution for patients that do not respond to conservative treatment for ED.

## Data Availability

The data supporting this study’s findings are available from the corresponding author upon reasonable request.
